# Behavioral moderators of *In-utero* superstorm sandy exposure and fronto-limbic cortical development—potential role of adaptiveness in clinical intervention strategies, a pilot study

**DOI:** 10.3389/fpsyt.2025.1481347

**Published:** 2025-07-17

**Authors:** A. Duke Shereen, Donato DeIngeniis, Tingting Wu, Md. Shafiur Rahman, Melissa Blum, Jeffrey H. Newcorn, Yoko Nomura

**Affiliations:** ^1^ Advanced Science Research Center at The Graduate Center, Neuroscience Initiative, City University of New York, New York, NY, United States; ^2^ Department of Psychology, Queens College, City University of New York, New York, NY, United States; ^3^ Department of Psychology, the Graduate Center, City University of New York, New York, NY, United States; ^4^ Beijing Key Lab of Learning and Cognition, School of Psychology, Capital Normal University, Beijing, China; ^5^ Graduate School of Health Innovation, Kanagawa University of Human Services, Kawasaki, Japan; ^6^ Research Centre for Child Mental Development, Hamamatsu University School of Medicine, Hamamatsu, Japan; ^7^ Department of Psychiatry, Icahn School of Medicine at Mount Sinai, New York, NY, United States; ^8^ Department of Epidemiology and Biostatistics, Graduate School of Public Health and Health Policy, City University of New York, New York, NY, United States

**Keywords:** natural disaster, prenatal stress, MRI, limbic, brain, behavior

## Abstract

**Introduction:**

Prenatal maternal stress may predispose a child to alterations in neurodevelopment and future psychopathology. Meanwhile, environmental disasters related to climate change are increasing in severity with significant impacts on physical and mental health. The current study explores the relationships among child behaviors, brain morphometry, and weather-related *in-utero* stress during Superstorm Sandy (SS).

**Methods:**

Parents completed the Behavioral Assessment System for Children, Second Edition (BASC-2) to quantify the extent of adaptive and clinical (externalizing/internalizing) behaviors at age 5. Magnetic resonance imaging of 9 SS-exposed and 21 non-exposed children at age 8 was used to assess brain volume. We analyzed main effects of *in-utero* SS exposure on brain volume/behavior and mediation-moderation models of exposure, behaviors and brain volume to determine how the association between exposure and brain volume is influenced by early childhood behavioral phenotypes.

**Results:**

The SS-exposed group had significantly greater externalizing behavioral problems, bilateral amygdala enlargement, and volumetric reduction of the left medial orbitofrontal cortex. While no behavioral phenotype mediated the association of exposure with brain volume, adaptive behaviors, as measured by four subdomains of the BASC-2 (social skills, activities in daily life, functional communication, and adaptivity), moderated the adverse impact of *in-utero* stress on brain volume later in life.

**Discussion:**

These findings highlight the importance of evaluating the interactive relationships among *in-utero* stress, behaviors, and neural development of the child to facilitate early identification and intervention for more vulnerable children. Promoting adaptive behaviors in early childhood may minimize the deleterious impact of prenatal stress exposure on subsequent brain development.

## 1 Introduction

Climate change poses an increasing threat to mental health. The growing frequency and intensity of hurricanes, tropical storms, wildfires, flooding, and droughts are disrupting social and economic stability and disproportionately endangering vulnerable populations such as pregnant women (1, 2). As a result of such environmental crises, prenatal maternal stress (PNMS), which broadly represents various forms of psychological distress during pregnancy (3, 4), has emerged as a target for intervention in recent years (5). Accumulating evidence demonstrates that elevated maternal anxiety and/or depressive symptoms during pregnancy are associated with child and adolescent *externalizing behaviors* such as elevated hyperactivity, aggression, and attention deficits, and *internalizing behaviors* such as elevated anxiety, depression, and somatic complaints (4, 6–17). However, more limited research has examined the role of *adaptive behaviors* on optimal child development (18). Adaptive behaviors refer to the behaviors and/or skill sets that are required for children to meet their basic needs for self-care, decision-making, communicating, and learning. More recent research shows that exposure to PNMS could facilitate adaptive behaviors, thereby acting as an inoculation to later life psychopathological outcomes (19). Various mechanisms and findings support this counterintuitive claim. Ceniceros et al. have shown maternal stress from temporary hospitalization-related relocation during pregnancy to lead to increased resilience in offspring observed from increased activity, decreased anxiety, increased interaction with novel objects, and increased temperamental confidence (20). Bondarenko et al. suggest a biological mechanism whereby PNMS leads to increases in placental and fetal serotonin levels which then mediate increased reactive and adaptive behaviors in offspring in mice studies (21). Several human studies (22–24) have shown that moderate levels of prenatal stress have an inoculation effect on developing infants, for example by being associated with advanced mental and motor skills, thereby promoting resilience (23, 25). However, neither the relationship between weather related *in-utero* stress and child brain development, nor potential positive influences of adaptive behaviors on promoting resilience among such populations, have been studied extensively.

Investigation of the underlying mechanisms linking maternal experiences during pregnancy to future child psychopathology has implicated dysregulation of the fetal hypothalamic-pituitary-adrenal (HPA)-axis, which in turn affects early neuronal development and leads to an increase in the offspring’s susceptibility to somatic diseases and mental health problems (3, 4). To evaluate the consequences of maternal stress on child neurophysiological development, magnetic resonance imaging (MRI) is often used for quantitative brain phenotyping. As the brain develops from the prenatal period through to infancy and early childhood, the limbic and prefrontal regions are at high risk of growth perturbations as they are most sensitive to stress exposure (26, 27). Further, the limbic and prefrontal regions form a highly interconnected circuit, allowing for coupling effects to spread alterations from one brain region to another, impacting related cognitive processes. For example, the amygdala has been shown to form extensive bidirectional connections with the orbitofrontal cortices, which in turn have been shown to influence emotion regulation and decision making (28, 29). The amygdala is a subcortical brain region functionally responsible for integrating sensory information and processing their emotional saliency. It is traditionally known as the “fight or flight” center of the brain and is involved in regulating the autonomic nervous and endocrine systems (30). Several disorders such as anxiety disorders, social phobias, intermittent explosive disorder, post-traumatic stress disorder, and panic disorders are linked to alterations in the amygdala and its connectivity to other brain regions. Key regions in the prefrontal cortex, such as the medial orbitofrontal cortex, are heavily implicated in regulating emotional behavior by projecting inhibitory connections to the amygdala (31). Connections between these regions develop rapidly in childhood and continue to mature into the third or fourth decade of life (32). Therefore, we focus the current study on the amygdala and medial orbitofrontal cortices.

Prior neuroimaging studies have aimed to pinpoint the structural and functional changes that occur in these brain regions to understand their relationship with behavioral changes among children exposed to prenatal stress (33). Interestingly, elevated stress has been found to be associated *with both decreased and increased* brain structure size. For example, some studies have reported a smaller amygdala volume among young adults exposed to elevated maternal stress during the first half of pregnancy (34), while others have reported a larger right amygdala in 4.5 year old girls exposed to elevated prenatal maternal depressive symptoms (35). In the frontal lobe, results also appear mixed. Heightened maternal anxiety during mid-gestation has been associated with reductions in gray matter density in the prefrontal cortex among child offspring, aged 6 to 9 (36). Reductions in the thickness of frontal and temporal cortical regions were also seen among child offspring born to mothers with depression (37, 38). In contrast, other studies have found increased cortical thickness in frontal regions to be associated with elevated prenatal maternal cortisol levels (39).

Brain structure has also been examined as a mediator between PNMS and child behaviors. Buss and colleagues reported that increased maternal cortisol levels during early gestation were associated with a larger right amygdala volume among girls at age 7, and amygdala volume was found to partially mediate the association between elevated maternal cortisol and affective problems (40). Similarly, Acosta and colleagues reported that elevated maternal anxiety during the second trimester was associated with a larger left amygdala volume among girls. Importantly, at 4 years of age, the volume of the left amygdala was found to partially mediate the association between maternal anxiety during pregnancy and child behaviors. However, contrary to the results of Buss et al, the partial mediation of a larger amygdala was related to a reduction, *not increase*, in child behavioral difficulties (41). In addition to the previous findings related to the amygdala, other work has demonstrated that prefrontal cortical thinning mediates the association between prenatal maternal depression and externalizing behaviors at around 8 years of age (42) as well as depressive symptoms during adolescence (43).

Given the complex and dynamic influences of the environment on brain and behavior, these seemingly contradictory findings are not entirely surprising, and may be due to any number of possible confounding and/or causal factors. For example, PNMS is quite general and can be subcategorized according to the source of the stressor, such as weather-related prenatal stress from natural disasters as is the case in the present study. It is also possible that associations between PNMS and neural development differ according to earlier behavioral problems or adaptive skills. However, to our knowledge, no study to date has examined both the possible mediating and moderating effects of behavioral expression earlier in childhood on associations between PNMS and subsequent neural development. While prior studies have examined the pathways from stress to later life behaviors via the indirect mediating path of neural indices, few have examined the predictiveness of behavior on later brain development. Here, we examine the role of clinical and adaptive behaviors both as potential *mediators* and *moderators* of brain morphometry as these behaviors may function through distinct developmental pathways: as mediators, these behaviors may explain the mechanisms by which prenatal stress exposure leads to changes in brain structure, while as moderators, the same behavioral patterns may influence the extent to which prenatal stress affects brain development. This dual analytical framework recognizes that both clinical symptoms and adaptive skills can serve as pathways of risk transmission and potential resilience factors.

Existing studies are also limited by temporal and etiological ambiguity surrounding the initiation and maintenance of PNMS. Measuring PNMS induced by exposure to isolated weather events, rather than from generalized psychological distress, allows for a less confounded identification of the cause and timeframe of the stressor. As an example, a quasi-experimental design paradigm explored the role of brain volume in the association between PNMS and child behavior by examining PNMS as a consequence of the 1998 Quebec Ice Storm. Among girls, an increase in left amygdala volume was found to partially mediate the positive association between prenatal maternal stress and externalizing behavioral problems. Similar results were found in the right amygdala among boys, prior to adjusting for postnatal factors (44). However, such quasi-experimental studies remain underrepresented in the literature on PNMS.

Here, we capitalize on existing behavioral data from the Stress in Pregnancy (SIP) study, a quasi-experiment with a longitudinal follow-up conducted by our group which focuses on the psychological consequences of exposure to Superstorm Sandy (SS), a large Category 3 hurricane which made landfall in Metropolitan New York in 2012 (45). To date, the SIP study has uncovered a trove of findings relating SS exposure to: clinical and adaptive behaviors (25), psychiatric disorders (46), suicidal ideation during the COVID-19 pandemic (47), changes in the placental transcriptome and infant temperament (48–50), and sex moderation of sympathetic nervous system with exposure (51). However, very little work has been done relating our behavioral findings to specific neuronal indices (52). The current work examines the relationship between PNMS, early childhood behavior, and brain development, with a specific focus on the mediating and moderating roles of early childhood behaviors on the association between *in-utero* SS exposure and later-life structural brain volumes of the bilateral amygdala and medial orbitofrontal cortex (mOFC). We address the following questions: 1) do SS-exposed children demonstrate different behavioral phenotypes (internalizing, externalizing, and adaptive behaviors) and fronto-limbic brain phenotypes compared to unexposed children?; 2) if so, are the effects of *in-utero* SS exposure on structural differences in brain development at age 8 directly or indirectly mediated by earlier-life clinical/adaptive behaviors?, and 3) do earlier-life behaviors moderate the relationship between *in-utero* SS exposure and brain structure? Elucidating the complex interplay of weather-related prenatal stress exposure and behavioral alterations in determining the trajectory of brain development will allow for early identification and intervention to enhance social and emotional resilience in children affected by natural disasters.

## 2 Materials and methods

### 2.1 Superstorm sandy exposure

The cohort was categorized into groups based on the timing of Superstorm Sandy exposure. Mothers of exposed children were pregnant at the time when Sandy made landfall, whereas the mothers of unexposed children were pregnant either before or after the storm (45).

### 2.2 Child behavior assessment

Child behaviors based on the Parent Rating Scale of the Behavioral Assessment System for Children, Second Edition (BASC-2) were reported by mothers for their school age children, at approximately age 5 (mean=4.51, SD=0.77, males/females=4.25/4.61, SD=0.36/0.86). For every item on the questionnaire, the child’s parent is asked to indicate if their child never, sometimes, often, or almost always performs a particular behavior. The BASC-2 is a well-standardized, multidimensional evaluation of the behavior of young children and measures both clinical (externalizing and internalizing problems) and adaptive (social skills, activity of daily living, functional communication, and adaptability) behaviors. Those scores were quantified using standardized T-scores (*mean*=50, *SD*=10), according to the BASC-2 system (53). Greater impairment is indicated by higher clinical (externalizing and internalizing) scores and lower adaptive scores (54). Internal consistency was excellent for the composite scales (all above α =.90).

### 2.3 MRI assessment

Participants were pooled from the Stress in Pregnancy (SIP) Study which has continued to follow children from the prenatal period to early adolescence since study inception in 2009 (45). Thirty children aged 5–11 years old (mean=8.50, SD=1.98, males/female=8.80/8.39, SD=1.54/2.14), participated in this pilot longitudinal study. Of those, 9 (30%) children were exposed (SS+) and 21 (70%) were not exposed to SS (SS-) *in-utero*. Despite the limitations of the small sample size, this pilot study can still inform future research and interventions given the initial findings and unique focus on adaptive behaviors. There were four siblings in the study (2 in SS+ and 2 in SS- groups). The protocol was approved by the Institutional Review Board at the City University of New York (CUNY). Exclusion criteria for participation in the parent study included HIV infection, maternal psychosis, maternal age <15 years, life-threatening maternal medical complications, and congenital or chromosomal abnormalities in the fetus. Exclusion criteria for MRI participation included metal implants, devices, and/or objects in the body.

### 2.4 MRI acquisition

All participants were scanned using a Siemens 3 Tesla Prisma MRI Scanner at the CUNY Advanced Science Research Center at approximately 8 years of age, several years after the child behavioral assessment. A mock scanner was used prior to imaging to acclimate children to the MRI environment. Children were taught to practice staying still by balancing a small toy on their nose, and the relationship between movement and good/blurry pictures was explained, similar to the “submarine protocol” (55). 3D high-resolution T1-weighted images were collected for each participant with a magnetization prepared rapid gradient echo (MP-RAGE) protocol with the following parameters: inversion time (TI)/repetition time (TR)/echo time (TE) = 1070/2500/2.9 msec, flip angle = 8.0 degrees, field of view = 256 mm × 256 mm, matrix size = 256 × 256, slice thickness = 1 mm without gap, and number of slices = 176. Real-time motion detection and correction was implemented using Volumetric Navigators (vNav) (56).

### 2.5 Brain tissue volume analysis

**Figure 1:**
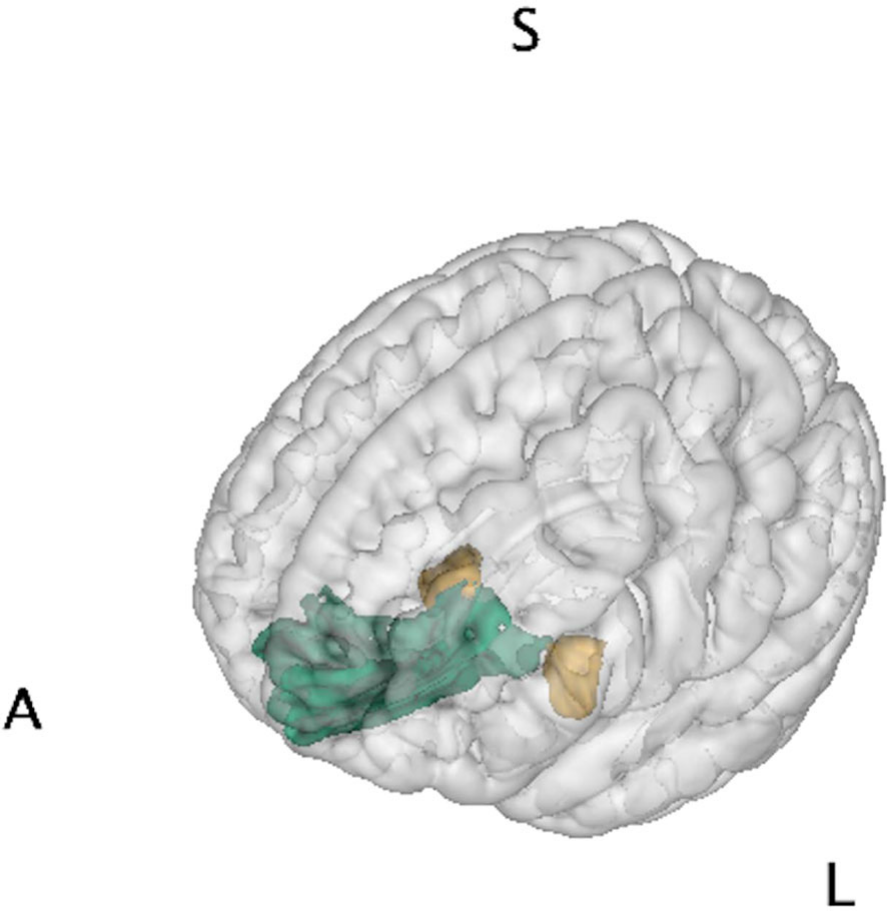
Brain regions of interest. Amygdala (yellow) and medial orbitofrontal cortex (green) were chosen as representative regions of the limbic system for brain-behavior analysis of Superstorm Sandy prenatal exposure. Letters denote orientation: A/S/L, anterior/superior/left.

The FreeSurfer pipeline was used to generate cortical and subcortical volumetric measures (57). The skull was stripped from the T1 images and the interface between the white and grey matter was estimated, which was further refined to obtain the thickness of grey matter. Cortical surfaces were inflated and Talairach transformation was performed. The cortex was parcellated into different anatomical regions using the Destrieux atlas. The brain regional volumes were normalized by the total intracranial volume on an individual basis. Analyses focused on the bilateral amygdala and mOFC defined by the atlas, illustrated in **Figure 1**.

### 2.6 Potential confounders

Child’s age, sex, objective SS challenges, and normative prenatal stress were considered *a priori* as potential confounders and were included in the models for statistical adjustment. Objective SS challenges were assessed by the Storm32 scale (46, 58) and normative prenatal stress was extracted using latent profile analysis (59) with pregnancy-related anxiety, depression symptoms, anxiety symptoms, perceived stress, and stressful life events. Normative prenatal stress was classified into three categories (low, medium, and high) (46).

### 2.7 Statistical approach

**Figure 2:**
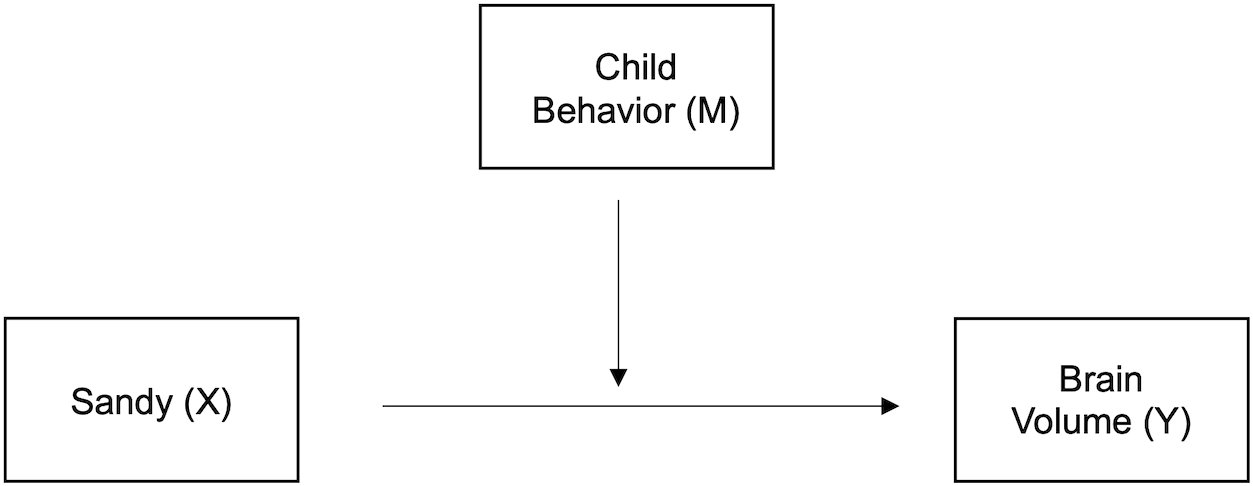
Moderation model. Early-life child behavior (externalizing/internalizing problems and adaptiveness) serves as the moderator, M, between Superstorm Sandy prenatal exposure, antecedent X, and brain volume during preadolescence, outcome Y.

First, tests of normality on all outcomes in relation to Superstorm Sandy exposure were conducted using QQ plots and Shapiro-Wilk tests. If the assumption of normal distribution was violated, appropriate normalization was applied. Initial diagnostics followed by a series of linear regressions via generalized estimating equations were conducted to determine group differences in behavioral or brain phenotypes and to model the influence of earlier life behaviors as potential moderators/mediators for the relationship between *in-utero* SS exposure and later life brain structure (**Figure 2**/**Supplementary Information**). For all analyses, potential confounders and intrafamilial correlations (i.e., four siblings) were adjusted. Finally, to correct for multiple testing, the Benjamini-Hochberg procedure was followed with a 15% false discovery rate (60) and applied to all analyses.

## 3 Results

### 3.1 Demographics

With the exception of child age at MRI assessment, there were no major demographic differences between the SS exposed and unexposed children (**Table 1**). The age at MRI assessment difference was expected, since to be considered SS exposed the child needed to be born during a very specific time frame.

**Table 1:** Demographic characteristics of participants by Superstorm Sandy exposure status.

Demographic variables	Total (n=30)	Superstorm Sandy Status		Statistics
		Exposed (n=9)	Not exposed (n=21)	
	Mean (SD), or n (%)			
Sex at Birth				*X* ^2^ (1)=1.59, *p*=.21
Female Male	22 (73.33) 8 (26.67)	8 (88.89) 1 (11.11)	14 (66.67) 7 (33.33)	
Child Age [BASC-2]	4.51 (.77)	4.24 (.75)	4.63 (.77)	*F* (1,28)=1.67, *p*=.21
Child Age [MRI]	8.50 (1.98)	6.64 (.98)	9.30 (1.75)	*F* (1,28)=18.04, *p*<.001
Maternal Race				*X* ^2^ (3)=1.43, *p* =.70
White	9 (30)	3 (33.33)	6 (28.57)	
Black	10 (33.33)	2 (22.22)	8 (38.10)	
Asian	1 (3.33)	0 (0)	1 (4.76)	
Multiracial	10 (33.33)	4 (44.44)	6 (28.57)	
Maternal Ethnicity				*X* ^2^ (1)=1.59, *p* = .21
Hispanic	22 (73.33)	8 (88.89)	14 (66.67)	
Non-Hispanic	8 (26.67)	1 (11.11)	7 (33.33)	
Maternal Martial Status				*X* ^2^ (2)=2.45, *p*=.29
Divorced/Separated	21 (70)	6 (66.67)	15 (71.43)	
Married	6 (20)	1 (11.11)	5 (23.81)	
Single	3 (10)	2 (22.22)	1 (4.76)	
Maternal Parity	2.90 (1.49)	2.67 (1.73)	3.00 (1.41)	*F* (1,28)=.31, *p=*.58
Family SES				*X* ^2^ (2)=2.46, *p =*.29
Low	1 (3.33)	0 (0)	1 (4.76)	
Medium	17 (56.67)	7 (77.78)	10 (47.62)	
High	12 (40)	2 (22.22)	10 (47.62)	
Maternal Substance Use				
Alcohol	1 (3.33)	1 (11.11)	0 (0)	X^2^ (1)=2.41, *p*=.12
Cigarette	7 (23.33)	3 (33.33)	4 (19.05)	*X* ^2^ (1)=.72, *p*=.40
Marijuana	10 (33.33)	4 (44.44)	6 (28.57)	*X* ^2^ (1)=.71, *p*=.40
Prenatal Stress				*X* ^2^ (2)=.37,*p*=.83
Low	9 (30)	2 (22.22)	7 (33.33)	
Medium	18 (60)	6 (66.67)	12 (57.14)	
High	3 (10)	1 (11.11)	2 (9.52)	
Objective Challenge^a^	2.45 (2.39)	2.67 (3.04)	2.36 (2.13)	*F* (1,28)=.10, *p*=.75
Maternal Age^b^	25.91 (6.36)	27.00 (6.91)	25.45 (6.23)	*F* (1,28)=.37, *p*=.55

NB: ^a^ number of objective challenges related to Superstorm Sandy within 3 months; ^b^assessed at the time of childbirth.

### 3.2 Is *in-utero* SS exposure associated with clinical/adaptive behaviors and amygdala/mOFC brain structural differences during early childhood?

**Figure 3:**
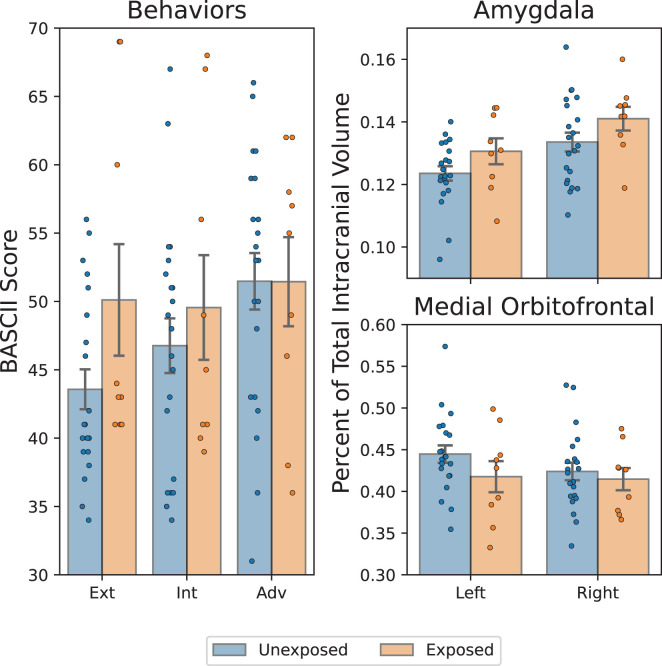
Comparison of *in-utero* hurricane exposure with childhood brain and behavior outcomes. Left: BASC-2 scores of externalizing/internalizing (ext/int) problems and adaptiveness (adv). Statistically significant greater externalizing problems among exposed group. Top/bottom right: amygdala/medial orbitofrontal cortex volume, expressed as a percentage of total intracranial volume. Significantly greater bilateral amygdala volume and significantly smaller right medial orbitofrontal cortex are present among exposed group: *p < 0.05, **p < 0.002, adjusted.

We examined the main effects of *in-utero* SS exposure on behaviors and brain volume (**Figure 3**). *In-utero* SS exposure was significantly associated with greater externalizing behaviors (*p* = .027), as we have previously found in the larger sample, indicating this subsample is relatively representative of the whole. No significant main effects were found for internalizing (*p* = .18) or adaptive behaviors (*p* = .39). *In-utero* SS exposure was associated with a larger left amygdala (*p* = .002) and right amygdala (*p* = .0016) as well as a smaller left mOFC (*p* = .013). No significant association was found between SS exposure group and the volume of the right mOFC (*p* = .42). Numerical results are summarized in **Table 2**.

**Table 2:** Brain and behavior differences between SS *in-utero* exposed and unexposed children.

Behaviors	SS(-)	SS(+)	Wald X^2^	*p*	*Adjusted* ^a^ *p*
	Mean (SE)	Mean (SE)			
Externalizing behavior	42.77 (1.15)	51.99 (3.38)	6.75	.009	**.027**
Internalizing behavior	45.74 (1.68)	51.93 (3.60)	2.43	.12	.18
Adaptive behavior	52.40 (1.83)	49.29 (3.04)	0.74	.39	.39
Brain volume	Mean (SE)	Mean (SE)	Wald X^2^	*P*	*Adjusted* ^a^ *p*
Left Amygdala	12.11 (0.18)	13.62 (0.41)	10.19	.001	**.002**
Right Amygdala	13.07 (0.21)	14.77 (0.38)	13.62	.00004	**.0016**
Left mOFC	45.16 (0.85)	40.09 (2.02)	6.02	.01	**.013**
Right mOFC	42.57 (0.95)	41.04 (1.74)	0.65	.42	.42

The model includes sex and age of the child, prenatal stress, and objective challenges related to the Superstorm Sandy event at the time of assessment. Intrafamilial correlation due to multiple children in a family has been corrected.

a Adjusted p-values with multiple-comparisons correction.

SS, Superstorm Sandy; mOFC, medial orbitofrontal cortex.

Bold values represent adjusted p-values less than 0.05.

### 3.3 Are structural differences in brain development between SS exposed and unexposed children at age 8 directly or indirectly mediated by earlier-life clinical and/or adaptive behaviors?

We observed statistically strong direct effects of SS exposure on all four hypothesized brain structures *except* the right mOFC. *In-utero* exposure to SS was associated with increased volume of the amygdala bilaterally (*p* < 0.0025) and decreased volume of the left mOFC (*p* < 0.013). However, we observed no statistically significant indirect mediating effects of any of the 3 behavioral measures on the association of SS exposure and either amygdala or mOFC volume. Therefore, while SS exposure significantly impacted regional brain volumes, this relationship was not dependent on the presence of externalizing, internalizing, or adaptive behaviors at age 5. **Supplementary Table S1** summarizes the mediation analysis results.

### 3.4 Do early-life child clinical and/or adaptive behaviors moderate the relationship between *in-utero* SS exposure and later-life brain structure?

Adaptive behaviors strongly moderated the relationship between SS exposure and all four brain structures we examined. Interestingly, the change in brain volume moderated by adaptive behaviors was opposite in the bilateral amygdalae (increased) compared to the bilateral mOFC (decreased). In stark contrast, neither clinical internalizing nor externalizing behaviors moderated the relationship between exposure and any of the brain structures of interest. Specific details with respect to each brain region are summarized below.

Amygdala: The association between SS exposure and left amygdala volume was moderated by adaptive behaviors (b = .14, *p* = .0005, i.e., better adaptive behaviors were associated with *greater* amygdala volume), but not externalizing (b = -.03, *p* = .36) or internalizing behaviors (b = -.02, *p* = .38). For the right amygdala, adaptive behaviors moderated the association (b = .08, *p* = .005, i.e., better adaptive behaviors were associated with *greater* volume), but not externalizing (b = -.02, *p* = .36) or internalizing (b = -.03, *p* = .40) (**Table 3**).

**Table 3:** Child behavioral phenotypes moderate the effect of SS exposure on brain volume.

Interactions	Left amygdala			Right amygdala			Left mOFC			Right mOFC		
	B (SE)	*p*	*adj p*	B (SE)	*p*	*adj p*	B (SE)	*p*	*adj p*	B (SE)	*p*	*adj p*
Sandy x EXT	-.03 (.05)	.13	.36	-.02 (.04)	.18	.36	.02 (.25)	.95	.95	-.03 (.21)	.90	.95
Sandy x INT	-.02 (.04)	.19	.38	-.03 (.03)	.30	.40	.36 (.19)	.07	.28	-.04 (.20)	.86	.86
Sandy x AS	.14(.04)	.0001.	**0005**	.08 (.03)	.003	**.005**	-.46 (.16)	.004	**.005**	-.31 (.13)	.02	**.02**

NB: The model includes sex and age of the child, prenatal stress, and objective challenges related to Superstorm Sandy as covariates. P-values reflect adjustment of interfamilial correlation. Adjusted p-values reflect adjustment of interfamilial correlation and multiple comparisons.

EXT, externalizing behaviors; INT, internalizing behaviors; AS, adaptive skills; mOFC, medial orbitofrontal cortex.

Bold values represent adjusted p-values less than 0.05.

mOFC: Similarly, the association between SS exposure and left mOFC volume was also moderated by adaptive behaviors (b = -.46, *p* <.005, however, unlike with the amygdala, adaptive behaviors were associated with *smaller* left mOFC volume), consistent with the direct effects of SS exposure (i.e., reducing left mOFC volume). In contrast, neither internalizing behaviors (b = .36, *p* = .28, nor externalizing behaviors (b = .02, *p* = .95) were significant moderators. The association between SS exposure and right mOFC volume was also moderated by adaptive behaviors (b = -.31, *p* = .02, i.e., better adaptive behaviors were associated with *smaller* right mOFC), but not externalizing (b = -.03, *p* = .95) or internalizing clinical behaviors (b = -.04, *p* = .86) (**Table 3**). This indicates a significant moderation effect of adaptive behaviors on right mOFC volume, even though the direct effects of SS exposure were not statistically significant.

## 4 Discussion

Our primary aims were to answer three central questions: 1) do SS exposed children exhibit marked differences in behavioral and brain phenotypes compared to unexposed children; 2) are effects of SS exposure on brain phenotypes mediated by previous behavioral phenotypes, and 3) do early-life behaviors moderate brain morphometry later in childhood? We have three major findings in parallel with these three primary aims. First, SS *in-utero* exposure alone predicts greater externalizing behaviors around age 5 as well as larger bilateral amygdala and smaller left mOFC around age 8. Second, none of the earlier life clinical and adaptive behaviors, including externalizing behaviors, mediated the relationship between SS exposure and later life brain structure in the fronto-limbic circuit, although this might have been predicted due to the greater externalizing behaviors observed in the SS-exposed group several years prior to structural brain measurements. Third, adaptive behaviors moderated the relationship between SS exposure and greater bilateral amygdala volume as well as smaller bilateral mOFC, while neither internalizing nor externalizing behaviors moderated any relationship between exposure and the brain structures of interest.

Perhaps counterintuitively, increased or decreased brain volume does not necessarily signal greater or poorer cognitive or behavioral function, respectively (61). Traditionally, the typical model has associated larger amygdala and smaller prefrontal cortices with increased disruptions in emotional regulation during development. This traditional model has been supported by findings from several studies, as examples: 1) an increased amygdala volume was found to underlie an increase in child problem behaviors, particularly anxiety and hypervigilance (i.e., internalizing problems) (33), 2) an enlarged right amygdala in girls has correlated positively with fearfulness in a cohort of healthy children and adolescents (62) and 3) asymmetrically increased amygdala and decreased orbitofrontal cortices have been associated with adolescent, male-specific aggressive behavior (63). While discrepancies in the direction of amygdala volume difference as a response to prenatal stress may be explained by sex differences, this model may oversimplify the interaction between environment, behavior, and brain by neglecting the many nuances and confounds in human development. Not surprisingly, many studies have presented findings that deviate from this traditional model (64). One alternative explanation overlooked in most studies, which we bring to light here, involves the role of resilience in the interplay of stress exposure, emotional regulation, and neurodevelopment.

Importantly, a child’s increased alertness to threat could serve as an adaptive mechanism to promote survival in the child’s stressful postnatal environment (3, 33). Similarly, impulsivity and aggression (i.e., externalizing behaviors) may help children cope better in more stressful postnatal environments. However, in a postnatal environment that is more benign than anticipated, the rise in child behavioral outcomes such as anxiety, impulsivity, and aggression are less adaptive. This stems from a mismatch between the life-threatening prenatal environment and the much less dangerous postnatal environment (3). Our results suggest that SS-exposed children may be more sensitive to perceived environmental threats, which likely contributes to their elevated externalizing phenotype and larger amygdala volume. In a similar vein, reductions in brain volume may not simply indicate poorer functioning, and in this case may be compensatory. The smaller mOFC volumes we observed in the SS cohort may relate to less hesitancy in responding to adverse stimuli, which could assist in situations of acute stress when it is necessary to make abrupt decisions to avoid an immediate perceived danger.

We hypothesize that this combination of increased amygdala and decreased mOFC volumes may facilitate greater resilience among children with prenatal SS exposure, relative to unexposed children. Here, resilience is defined as an adaptive response to serious hardship; it is rooted in evolution-based theories which posit that individuals better respond to stress via effective coping and adaptive strategies. Resilience manifests in demanding situations, including periods of collective adversity, such as the aftermath of a natural disaster (65). While transient activation of the stress system as seen in the manifestation of externalizing and internalizing behaviors may be protective against highly threatening environments, we are not certain if this will be a long-lasting advantage beyond mid-childhood when the cohort’s brain volumes were measured. Severe exposure to stress has been suggested to leave the brain “stuck” in a state of high alert, causing chronic strain. Chronic strain leads to wear and tear on the brain and body later in development, a concept known as allostatic load (26, 61). As such, short-term survival benefits, through elevated vigilance and stress reactivity (i.e., clinical behaviors in this study), may come at a later cost due to the future risk of poor neurodevelopmental outcomes (3). Such long-term costs may include both physical, i.e. increased heart rate and blood pressure, and mental, i.e. risk of developing post-traumatic stress disorder, memory and attention deficits, increased irritability and mood swings.

It is important to recognize, however, that adaptive behaviors are also responses to stress. Obviously, adaptive behaviors act on the allostatic load, but in an opposite direction from the clinical (externalizing and internalizing) behaviors, contributing to greater resilience. Unlike these behaviors, adaptive behaviors may operate differently and affect different or additional parts of the brain. Our finding related to the contrasting role of adaptive behaviors is novel: adaptive behaviors moderate the effects of SS-stress on neural development. It is also important to note that our cross-sectional design does not logically imply a causal link between adaptive behaviors and protection from neurodevelopmental risk. However, since the promotion of adaptive, or resilient, behaviors in the face of traumatic exposures is often neglected, despite its importance in alleviating the impact of stress, it has great public health implications.

While we found early-life adaptive behaviors moderated the association between SS exposure and an enlarged/reduced amygdala/mOFC volume, it is possible that increased adaptive skills and fronto-limbic alterations may leave individuals more sensitive to later life experiences, with the potential to both exacerbate and mitigate the effects of a negative environment. This emphasizes the importance of continuing to assess the same group of children over time to examine the trajectories of brain development and to clarify the complex interplay between behaviors and brain development. It is important to note that alterations found in brain development from early-life adversity may not be permanent. For example, initial enlargements found in the amygdala from early-life adversity may eventually lead the amygdala to become sensitized to future stressors, leading to volume reductions during adulthood (27). More research needs to be conducted to gain a more complete understanding of the short and long-term implications of early-life exposure to adversity on neurobehavioral development and emotion regulation. In the short term, it seems clear from our cohort that an orientation in clinical focus towards identifying and strengthening adaptive behaviors may have a tremendous positive effect.

Several prior studies have suggested potential intervention strategies for targeting adaptive behaviors with a strong focus on children diagnosed with Autism Spectrum Disorder (ASD), a population known to display deficits in adaptive skills (66). These strategies include video modeling for teaching social skills (67), functional communication therapy for reducing severe problem behaviors (68), and cognitive behavioral therapy (69) for increasing daily living skills in children with high-functioning ASD and comorbid anxiety symptoms. While our sample primarily presents with subclinical symptoms, the findings from this research can inform the development of targeted therapies for adaptive behaviors among prenatally stress-exposed children. Approaches that strengthen family relationships, build emotional regulation skills, communication skills, and enhance coping strategies would appear to be particularly relevant based on our results. Additionally, interventions that address both individual and family-level factors that embrace environments promoting adaptive skills and provide children with scaffolding may be most effective for promoting resilience and adaptive functioning. However, these preliminary findings require replication in larger samples before specific clinical recommendations can be made.

### 4.1 Limitations, strengths, and conclusions

The study is based on a small sample size (N=30). Of these, only 9 children were exposed to Superstorm Sandy, thereby limiting robust generalizations from the statistical comparisons and potentially introducing biases. Thus, findings were limited to group differences and were not sufficiently powered to explore finer subdivisions by sex or trimester-specific exposure. While we controlled for highly pertinent potential confounders (child age and sex) as well as various prenatal confounders, such as normative prenatal stress and severity of SS challenges, there are other possible confounders. For example, although all mothers of the SS exposed group were pregnant and in the New York area during the hurricane, some experienced greater hardship and more stress than others. Another potentially important confounder is maternal mental health, which may have a strong influence on caregiver quality. Since our sample size prevents us from incorporating a wider array of confounders, future work that includes the overall cohort (N=~350 mother-child dyads) will help resolve these limitations. An additional limitation is a potential reporting bias in behavioral measures as they relied on maternal reporting. Also, the false discovery rate threshold choice of 15%, while appropriate for hypothesis-generating research and to reduce Type II error, may limit the definitiveness of our conclusions. Lastly, we do not have brain imaging data at age 5 when behavioral phenotypes were measured. The absence of this data limits confidence with which we can connect brain structure and behavioral phenotype data cross sectionally. Caution is warranted to prevent over-generalization of these findings. Having multiple assessments of the brain imaging and behavioral phenotype data could have helped elucidate the relationship more clearly. Furthermore, additional investigation of functional imaging analysis would further help clarify the possible interplay of these regions within a broader brain network. It is also possible that neural development at age 5 could have moderated the associations between weather-related *in-utero* stress and later behavioral problems, although it would be naïve to assume that the brain structures of interest did not change in the critical years of development between five and eight. Future studies should include concurrent behavioral assessments with neuroimaging to better understand brain-behavior relationships. Still, it is clear that early behavioral indicators can be used to identify a subsequently measured pattern of altered neural development. The study also has notable strengths. First, the cohort has been followed since birth and behavioral assessments were blinded with respect to brain volume measurements. Second, our findings are robust to adjustment for multiple testing, the intrafamilial correlation due to 4 siblings’ participation, and various pertinent confounders, such as sex and age as well as different sources of prenatal stress, in all analyses. We will also continue to follow these children to gain greater insight into the current findings.

### 4.2 Future directions

Several key research directions emerge from this pilot study. First, longitudinal follow-up studies are needed to track neurodevelopmental trajectories over time and understand how prenatal stress, adaptive behaviors, and brain development interact across childhood and adolescence. Second, future expansions to larger and more diverse samples will improve reliability and enable sex-specific and trimester-specific analyses that our current sample could not examine. Third, intervention-based research should test whether promoting adaptive behaviors can mitigate prenatal stress effects on brain development, providing clinical value by identifying modifiable protective factors. These directions will guide future research and help translate our preliminary findings into clinically meaningful interventions for at-risk children.

### 4.3 Conclusion

We measured weather-related *in-utero* stress using a quasi-experimental design where SS exposure served as a naturally assigned independent variable. This distinguishes our analysis from many other studies, which tend to measure PNMS by more subjective methods, such as self-reported scales. However, the small sample size, particularly the SS exposed group, is a significant limitation and warrants conservative interpretation of the results. It is important to note that our composite adaptive behaviors scale was measured by four subscales that identify the level of child adaptation skills, including skills in daily living activity, social skills, functional communication, and adaptability. As such, it is critical to inform policy makers, educators, and pediatricians of the moderating role that adaptive behaviors can play in potentially ameliorating the deleterious effects of disaster-induced PNMS on amygdala and mOFC volume. In summary, our findings will help inform early identification and intervention strategies that can target specific behavioral phenotypes, especially to promote resilience and to minimize the risk of altered brain development among children exposed to prenatal stress.

## Data Availability

The raw data supporting the conclusions of this article will be made available by the authors, without undue reservation.
