# Supplementary material for: Behavioral moderators of In-utero superstorm sandy exposure and fronto-limbic cortical development—potential role of adaptiveness in clinical intervention strategies, a pilot study
Source: Front Psychiatry. 2025 Jul 17;16:1481347. doi: 10.3389/fpsyt.2025.1481347 (PMC12312225; doi:10.3389/fpsyt.2025.1481347)
Supplement: Supplementary file 1 [file Table1.docx]

**Supplemental Information**

In addition to the main moderation analysis, we tested for indirect effects of behavior mediation on brain structural development for completeness. While our direct effects were strong and corroborate our main effects findings (Table 2), none of the indirect effects were near significance (Table S1). It is not clear whether to expect a null result as behavioral phenotypes were assessed years before MRI brain structural measurements (i.e., changes in brain structure have influence over behavior, but not vice versa during the time scales we made our observations) or that the sample size is simply not large enough to provide sufficient statistical power. Future work with longitudinal data in a larger sample size and over larger time periods may help elucidate these questions related to possible mediation influences.

**Table S1 Mediation of Behavioral Phenotypes on SS Exposure and Brain Volume at age 9**

| Paths | **Hemisphere** | **Direct effects** | | **Indirect effects** | | **Total effects** | |
| --- | --- | --- | --- | --- | --- | --- | --- |
|  |  | **B (95% CI)** | ***Adj^a^ p*** | **B (95% CI)** | ***Adj^a^ p*** | **B (95% CI)** | ***Adj^a^ p*** |
| Sandy 🡪 Ext 🡪 Amygdala | Left | 1.839 (0.872,2.807) | <0.001 | -0.327 (-0.812,0.159) | 0.187 | 1.513 (0.556,2.469) | 0.004 |
|  | Right | 1.791 (0.645,2.937) | 0.002 | -0.113 (-0.483,0.257) | 0.550 | 1.678 (0.74,2.616) | <0.001 |
| Sandy 🡪 Int 🡪 Amygdala | Left | 1.574 (0.713,2.435) | <0.001 | -0.061 (-0.623,0.5) | 0.831 | 1.513 (0.556,2.469) | 0.004 |
|  | Right | 1.688 (0.701,2.675) | 0.0024 | -0.010 (-0.105,0.086) | 0.845 | 1.678 (0.74,2.616) | <0.001 |
| Sandy 🡪 AS 🡪 Amygdala | Left | 1.546 (0.611,2.481) | 0.0024 | -0.033 (-0.175,0.108) | 0.644 | 1.513 (0.556,2.469) | 0.004 |
|  | Right | 1.632 (0.636,2.627) | 0.0024 | 0.046 (-0.151,0.243) | 0.644 | 1.678 (0.74,2.616) | <0.001 |
| Sandy 🡪 Ext 🡪 mOFC | Left | -5.777 (-9.815,-1.738) | 0.0068 | 0.670 (-1.109,2.45) | 0.460 | -5.106 (-9.032,-1.18) | 0.013 |
|  | Right | -2.588 (-6.782,1.606) | 0.271 | 0.900 (-0.919,2.718) | 0.332 | -1.688 (-5.405,2.028) | 0.373 |
| Sandy 🡪 Int 🡪 mOFC | Left | -5.170 (-9.169,-1.171) | 0.013 | 0.064 (-0.585,0.713) | 0.848 | -5.106 (-9.032,-1.18) | 0.013 |
|  | Right | -1.794 (-5.757,2.168) | 0.375 | 0.106 (-0.866,1.078) | 0.831 | -1.688 (-5.405,2.028) | 0.373 |
| Sandy 🡪 AS 🡪 mOFC | Left | -5.501 (-9.292,-1.71) | 0.004 | 0.395 (-1.027,1.816) | 0.586 | -5.106 (-9.032,-1.18) | 0.013 |
|  | Right | -2.048 (-5.481,1.385) | 0.264 | -0.327 (-0.812,0.159) | 0.187 | -1.688 (-5.405,2.028) | 0.373 |

Ext = Externalizing Problems; Int = Internalizing Problems; AS= Adaptive Skills

Sandy= *in-utero* exposure to Superstorm Sandy; L= left; R= right; mOFC = medial orbitofrontal cortex

Age and sex of the participant, prenatal stress, and objective challenges related to Superstorm Sandy exposure have been adjusted in the model. Intrafamilial correlation due to multiple children in a family has been corrected.

1. Multiple comparisons have been adjusted.
